# Hyaluronic Acid-Coated Chitosan/Gelatin Nanoparticles as a New Strategy for Topical Delivery of Metformin in Melanoma

**DOI:** 10.1155/2023/3304105

**Published:** 2023-06-05

**Authors:** Pedram Ebrahimnejad, Anahita Rezaeiroshan, Amirhossein Babaei, Azin Khanali, Shaghayegh Aghajanshakeri, Ali Farmoudeh, Ali Nokhodchi

**Affiliations:** ^1^Department of Pharmaceutics, Faculty of Pharmacy, Mazandaran University of Medical Sciences, Sari, Iran; ^2^Pharmaceutical Sciences Research Center, Hemoglobinopathy Institute, Mazandaran University of Medical Sciences, Sari, Iran; ^3^Ramsar Campus, Mazandaran University of Medical Sciences, Ramsar, Iran; ^4^Department of Toxicology and Pharmacology, Faculty of Pharmacy, Mazandaran University of Medical Sciences, Sari, Iran; ^5^Pharmaceutics Research Laboratory, School of Life Sciences, University of Sussex, Brighton, UK; ^6^Lupin Research Center, Coral Springs, FL, USA

## Abstract

Metformin is a multipotential compound for treating diabetes II and controlling hormonal acne and skin cancer. This study was designed to enhance metformin skin penetration in melanoma using nanoparticles containing biocompatible polymers. Formulations with various concentrations of chitosan, hyaluronic acid, and sodium tripolyphosphate were fabricated using an ionic gelation technique tailored by the Box-Behnken design. The optimal formulation was selected based on the smallest particle size and the highest entrapment efficiency (EE%) and used in *ex vivo* skin penetration study. *In vitro* antiproliferation activity and apoptotic effects of formulations were evaluated using MTT and flow cytometric assays, respectively. The optimized formulation had an average size, zeta potential, EE%, and polydispersity index of 329 ± 6.30 nm, 21.94 ± 0.05 mV, 64.71 ± 6.12%, and 0.272 ± 0.010, respectively. The release profile of the optimized formulation displayed a biphasic trend, characterized by an early burst release, continued by a slow and sustained release compared to free metformin. The *ex vivo* skin absorption exhibited 1142.5 ± 156.3 *μ*g/cm^2^ of metformin deposited in the skin layers for the optimized formulation compared to 603.2 ± 93.1 *μ*g/cm^2^ for the free metformin. Differential scanning calorimetry confirmed the deformation of the drug from the crystal structure to an amorphous state. The attenuated total reflection Fourier transform infrared results approved no chemical interaction between the drug and other ingredients of the formulations. According to the MTT assay, metformin in nanoformulation exhibited a higher cytotoxic effect against melanoma cancer cells than free metformin (IC_50_: 3.94 ± 0.57 mM vs. 7.63 ± 0.26 mM, respectively, *P* < 0.001). The results proved that the optimized formulation of metformin could efficiently decrease cell proliferation by promoting apoptosis, thus providing a promising strategy for melanoma therapy.

## 1. Introduction

The skin is the biggest organ in the human body and is the first crucial barrier against various diseases [1]. It is reported that skin cancer is prevalent in the United States of America, and about one out of five citizens is challenged with this malignancy [2]. Melanoma is a form of skin cancer that indicates a complex mutagenic disorder in melanin cells (melanocytes) and is among the five most prevalent cancers with increased mortality percent. [3, 4]. Some invasive treatment strategies such as surgery, chemotherapy, and immunotherapy are available nowadays. However, chemotherapy can cause damage to normal cells and may fail because of drug resistance when two or three anticancer agents are utilized [5]. Metformin hydrochloride (MET) is a biguanide mainly used to treat diabetes type II mellitus, and it is also mentioned as a required drug for other diseases [6, 7]. New studies showed that MET could be effective along with other chemotherapy drugs in cancer treatment [8]. The anticancer effect of MET might be due to the activation of the AMP-activated protein kinase (AMPK) route, which makes interventions in protein synthesis and may cause death to melanoma cells and inhibit tumor growth [8]. In the biopharmaceutical classification system (BCS), MET is categorized as a class III drug with high water solubility and low permeability to the cell membrane. Its log *P* is −1.43 and pKa value is 11.5 [9, 10]. The utilization of this drug through epithelial cells is limited due to its high solubility in water. It is considered that delivering a drug with such characteristics to the skin layers is a major challenge. In order to diminish this problem, the application of biodegradable polymers was involved [11].

Lately, nanotechnology research has revealed that colloidal systems are beneficial for targeting active ingredients to specific organs, decreasing adverse effects, and improving treatment efficacy [12–14]. Due to their therapeutic features, various nanocarriers gained lots of attention, including liposomes, polymeric nanoparticles, dendrimers, and gold nanoparticles [5].

Chitosan (CHI) and gelatin are natural polymers found in the tissues of living organisms, especially plants and animals. They are extensively used in pharmaceutical drug delivery systems because of their noncytotoxic, biocompatible, and biodegradable nature [15–19]. The polymers can form colloidal particles that coat the active ingredient and lead the drug to pass through the skin layers [20–23]. Since both CHI and gelatin have good health-promoting properties, it is anticipated that the combination of these two polymers may serve as a promising matrix for transdermal delivery of drugs with improved healthcare. Recently, hyaluronic acid (HA), a natural polysaccharide, has been functionalized in drug delivery as a ligand because of its binding ability to receptors such as CD44 in both normal and cancerous cells and facilitates drug aggregation in cancerous cells. HA has shown astonishing efficacy as a carrier for anticancer and gene delivery [24].

Although there are several studies on the targeted delivery of MET, no reports have been found to explore the topical delivery of MET using polymeric nanoparticles against melanoma. To this end, the present investigation involved the preparation of nanoparticles of chitosan and gelatin with HA networks loaded with MET (MET-loaded NPs) as a model drug followed by *in vitro* transdermal delivery evaluation of the formulations using a Franz diffusion cell. In addition, the effect of each parameter was evaluated by response surface methodology (RSM). Accordingly, the main aim of the current study is to enhance the efficiency of metformin nanoparticles through transdermal delivery to treat melanoma.

## 2. Materials and Methods

### 2.1. Materials

Metformin was purchased from Tehran Chemie Pharmaceuticals Co. (Tehran, Iran). Chitosan with low molecular weight (deacetylation ≥ 75%) was purchased from Fluka (Buchs, Switzerland). Hyaluronic acid (MW 200-400 kDa) was purchased from Contipro (Dolní Dobrouč, Czech Republic). Gelatin type B (bovine skin with bloom ~225), sodium tripolyphosphate (STPP), penicillin-streptomycin antibiotic, and 3-[4,5-dimethylthiazol-2-yl]-2,5-diphenyltetrazolium bromide (MTT) were supplied from Sigma-Aldrich (St. Louis, MO, USA). Phosphate buffer saline (PBS, pH 7.4), trypsin-EDTA, fetal bovine serum, and Dulbecco's Modified Eagle's Medium (DMEM) were purchased from Gibco-BRL (Grand Island, NY, USA). Deionized water (DW) was obtained from Human Ultra-Pure System (Human Corp., Korea). Ethanol, acetic acid, and all other chemicals utilized were of analytical grade and were supplied from Merck KGaA (Darmstadt, Germany).

### 2.2. Box-Behnken Design (BBD)

RSM is used as a mathematical technique to match experimental information with a statistical model to achieve the optimal formula. RSM followed by BBD with three independent variables (CHI, STPP, and HA concentrations (mg/ml)), three levels (-1, 0, and 1), and 15 formulations was applied to investigate the relations between independent variables and the responses [25]. The polynomial model, which explains the relations between the variables, is presented in the following equation:

(1) $$\begin{array}{c} Y=\beta_{0}+\beta_{1}A+\beta_{2}B+\beta_{3}C+\beta_{12}AB+\beta_{13}AC+\beta_{23}BC+\beta_{11}A^{2}+\beta_{22}B^{2}+\beta_{33}C^{2}, \end{array}$$

where *A*, *B*, and *C* are the independent variables. The measured response associated with each factor was denoted by *Y*. The coefficients of the polynomial equation were denoted as *β*_0_ (intercept); *β*_1_, *β*_2_, and *β*_3_ (linear effects); *β*_11_, *β*_22_, and *β*_33_ (quadratic effects); and *β*_12_, *β*_13_, and *β*_23_ (interaction effects). The interaction and quadratic terms are represented by the words *AB*, *BC*, *AC*, *A*^2^, *B*^2^, and *C*^2^. The various ratios of variables, their levels, and the responses are demonstrated in Table 1.

### 2.3. Preparation of Polymeric Nanoparticles

Nanoparticles (NPs) were prepared using the modified ionic gelation method based on different electric charges between polymers [26]. First, CHI was dissolved in 10 ml of 1% (*v*/*v*) acetic acid solution and placed in the bath sonicator (Pars Nahand, Iran) for 15 minutes. Then, this solution was stirred for half an hour and hydrated for 24 hours at 25°C. Next, a constant amount of gelatin (5 mg) was dissolved in 5 ml of deionized water at 60°C and sonicated for 20 minutes. The obtained gelatin solution was thoroughly mixed with the CHI solution, and then, MET (10 mg) was added. The mixture was stirred at 300 rpm for 1 hour. According to Table 2, specific amounts of HA and STPP were dissolved in water. The latter solution was added to the drug-containing polymer solution. Finally, the pH of the final suspension was adjusted to 5.5 with sodium hydroxide solution (1 M), and then, the NPs were stirred for one hour. The MET-loaded NPs were separated by ultracentrifugation at 13,000 rpm for 20 minutes and then reconstituted in DW. The resultant suspension was frozen and lyophilized for 24 hours at -50°C using a freeze dryer (Alpha 11-2 LDplus, Martin Christ, Germany). Lyophilized powder was kept at 4°C until used in a subsequent study. To prepare non-HA@MET-loaded NPs, the above steps were performed, with the point that HA was not used during production.

### 2.4. Optimization of Formulation Employing Box-Behnken Design

As BBD is an effective way to quickly optimize a process, therefore, MET-loaded NPs were optimized using BBD. To adapt the replies to the proper mathematical model produced by design, many statistical measures such as the probability value (*P* value), the regression coefficient (*R*^2^ value), the Fisher model value (*F* value), and the lack of fit *F* value were utilized. Quadratic polynomial response equations, including key components and interaction factors, have been created in addition to the best-fit model. An ANOVA was employed to evaluate the appropriateness and validity of the provided model. The best formulation was chosen based on the desirability of the response variable.

### 2.5. Characterization of the Prepared NPs

A dynamic light scattering (DLS) experiment was employed to determine the size, zeta potential, and PDI (polydispersity index) of MET-loaded NPs (Zetasizer Nano ZS system, Malvern Instruments, Worcestershire, UK) [27]. The angle of detection was 90°, the temperature was 25°C, the concentration of the specimens was 20–400 kilo counts per second, and the intensity of diffraction was 100 kilo counts per second.

### 2.6. Evaluation of Entrapment Efficiency (EE%)

The centrifugation technique was selected to determine the percentage of entrapment efficiency. The NPs were separated using ultracentrifuge at 13000 rpm for 20 minutes at 4°C using an ultracentrifuge (Sigma 3-30KS, Germany) to separate the MET-loaded NPs from the dispersion [28]. The supernatant was detached to determine the amount of unentrapped MET by UV spectrophotometry (230 nm). EE% was calculated as follows [29]:

(2) $$\begin{array}{c} \text{Entrapment Efficiency }\left(\text{EE}\%\right)=\frac{\text{Total drug}-\text{drug in supernatant}}{\text{Total drug}}\times 100. \end{array}$$

### 2.7. Differential Scanning Calorimetry (DSC)

The freeze-dried powder of MET-loaded NP optimized formulation and pure ingredients (CHI, gelatin, MET, STPP, and HA) was covered in aluminum pans. Samples were analyzed by Pyris 6 calorimeter (Perkin Elmer, Netherlands) between 30°C and 300°C and a scanning speed of 10°C/min [30].

### 2.8. Attenuated Total Reflection-Fourier Transform Infrared (ATR-FTIR) Analysis

In order to verify interactions between the components of the optimized formulation (CHI, gelatin, MET, STPP, and HA), ATR-FTIR analysis was carried out using Cary 630 (Agilent Technologies Inc., CA, the United States). Each spectrum was registered between 4000 and 650 cm^−1^, with a resolution of 1 cm^−1^. All the components of the MET-loaded NP optimized formulation, along with the pure drug, went under this investigation [31].

### 2.9. Morphology Investigation

Scanning electron microscopy (SEM) was applied to exhibit the morphology of nanoparticles (size and shape) in the optimized formulation by FEI Quanta 200 (FEI Company, USA). Gold was utilized to coat the samples, and morphology was reported [32].

### 2.10. Cetyl Trimethyl Ammonium Bromide (CTAB) Turbidimetric Analysis

The amount of HA in the NPs was determined by an indirect method using a CTAB turbidimetric assay. After centrifugation, the quantity of free HA in the supernatant was determined. The quantity of encapsulated HA was estimated by subtracting the supernatant amount from the initial amount utilized in the preparation. In brief, 50 *μ*l standard solutions of HA (0.05-0.6 mg/ml) were included in the ELISA 96-well plate. Fifty microliter phosphate buffer (pH 5.5) was added to each well and incubated for 10 minutes at 37°C. CTAB solution (10 *μ*M and 100 *μ*l) was included in the wells and was read through for 10 minutes at 570 nm utilizing a microplate reader (ELx800, BioTek, Winooski, VT, USA). In order to evaluate HA in the optimized formulation, the supernatant was removed by centrifugation. Instead of the standard solution, the supernatant was examined. Each step was performed three times [33].

### 2.11. *In Vitro* Release Assessment

To evaluate the release pattern of the MET-loaded NPs and drug solution, the dialysis bag technique was used. In brief, 5 ml of the formulation was transferred to a dialysis tube, capped, and immersed in 25 ml of phosphate buffer pH 5.5 due to MET hydrochloride's propensity to be more soluble in an acidic medium [34] while being shaken at 100 rpm at 32 ± 0.5°C. At predetermined times (0.5, 0.75, 1, 2, 4, 6, 8, 12, and 24 h), 2 ml of the dissolution medium was removed and restored with the same volume of fresh medium to keep the sink condition [26, 32]. The MET concentration in each sample was determined by UV spectroscopy. The in vitro drug release data were fitted to kinetic models (zero-order, first-order, Higuchi, and Korsmeyer–Peppas). The model with the highest determination coefficient (*R*^2^) and the lowest root mean square error (RMSE) was selected to describe the drug release mechanism. KinetDS 3.0 software (Jagiellonian University Medical College, Poland) was used to perform kinetic calculations.

### 2.12. *Ex Vivo* Skin Permeation and Retention Study

Examination of animals for skin permeation study was followed by Ethical Guidelines for Investigations in Laboratory Animals. The Ethics Review Committee of Mazandaran University of Medical Sciences (MAZUMS) approved animal experimentation. Male Wistar rats between 170 and 200 g were sacrificed, and after approval of their death, the abdominal skin was shaved and removed. The full-thickness skin was soaked in an isotonic saline solution 24 h before the experiment. The Franz diffusion cell technique investigated MET skin permeation from the optimized formulation and free drug solution. Briefly, 2 ml of the formulation was placed in the donor chamber. The receptor chamber was filled with phosphate buffer (pH 5.5 at 32 ± 0.5°C) and stirred at 150 rpm by a magnetic stirrer. The acidic pH of the skin surface has been identified as a regulatory element in the maintenance of stratum corneum (SC) barrier permeability [35]. Because the usual pH of the skin is about 5.5, a phosphate buffer with a pH of 5.5 was chosen for the *in vitro* skin investigation [36]. At predetermined times (0.5, 1.5, 3, 6, 12, and 24 h), 2 ml of the receptor medium was collected and quantified using a UV spectrophotometer. The same volume of the fresh medium was returned to the cell [37]. As a blank for each experiment, three Franz cells were placed. The Franz cells with blank NPs were handled similarly to the other cells, except that no drug was placed into the exposure chamber [38]. The limit of detection (LOD) and limit of quantitation (LOQ) were 1.24 *μ*g/ml and 3.75 *μ*g/ml, respectively, while the detection range was 2–20 *μ*g/ml. For assessing the skin integrity, methylene blue dye was used [39]. After the completion of the permeation study, 0.5 ml of a 0.025% methylene blue aqueous solution was applied to the skin for 30 minutes before being washed with DW. In the case of any dye passage before digestion and processing, the skin was considered altered skin. A spectrophotometer was employed at 661 nm to evaluate the receptor compartment for dye permeation. The skins were removed when the permeation experiment was finished, washed three times with DW to remove the residual formulations and dye, and dried to find out how much MET remained within the skin. The tissue was then divided into tiny pieces with surgery scissors and placed in a clean beaker filled with DW. The beaker was then sonicated with a bath sonicator for one hour to make the skin sections digest. The supernatant was finally filtered using filter paper followed by a syringe filter with a 0.22 *μ*m pore size. The amount of MET in the sample was measured using a UV spectrophotometer.

### 2.13. *In Vitro* Cytotoxicity Assay

The *in vitro* cytotoxicity of MET-loaded NPs on human melanoma (A375) cancer cells was estimated by MTT (3-[4,5-dimethylthiazol-2-yl]-2,5-diphenyltetrazolium bromide) assay which was slightly modified from prior work [40]. The Pasteur Institute supplied a cell line (Tehran, Iran). Cells were cultured in DMEM supplemented with 10% fetal bovine serum and 1% penicillin-streptomycin solution in a 5% CO_2_ condition with a relative humidity of 95%. Cells were seeded at a density of 1 × 10^4^ cells/well in a growth medium in a 96-cell microplate and incubated for 24 h to perform the cytotoxicity experiment. Cells were treated with a serial dilution of MET ranging from 0.5 to 8 mM for MET-loaded NPs, with wells treated with an equal quantity of free MET solution or blank NPs as comparisons. After incubation for 24 h, cells were given 20 *μ*l of 2.5 mg/ml MTT in PBS and incubated for another 4 hours at 37°C. The MTT solution was then withdrawn entirely, and 100 *μ*l DMSO was applied to dissolve the precipitate. An ELx800 microplate reader was used to detect absorbance at 570 nm (BioTek, Winooski, VT, USA). Cell viability (%) at different concentrations was calculated as (optical density (OD) of treated cells/OD of control cells) × 100. All experiments were repeated 3 times.

### 2.14. Annexin V/Propidium Iodide Double Staining Assay

The assessment of cell death following treatment of A375 cells with the mentioned formulations (MET-loaded NPs, blank NPs, and free MET) was carried out by labeling the cells with an Annexin V-FITC and propidium iodide (PI) solution, followed by flow cytometry analysis. Briefly, A375 melanoma cells were placed in a 6-well culture plate at a density of 5 × 10^5^ cells/well. The cells were subjected to all formulations at each group's IC_50_ value (as obtained in the MTT assay) after 24 hours. The cells were trypsinized after being incubated for 48 hours, centrifuged for 5 minutes at 2000 rpm, and then washed twice with PBS and redispersed in Annexin V binding buffer. After that, the samples were treated with 10 *μ*l of Annexin V-FITC labeling solution and 5 *μ*l of the PI solution, followed by incubation for 5 min at 25°C in the dark. Finally, the stained cells (with Annexin V-FITC and PI) were analyzed by flow cytometry (FACS Calibur, USA). The negative control for each test group determined the quadrant gate (unstained sample).

### 2.15. Statistical Analysis

The mean ± standard deviations (SD) of three experiments were used to present all of the results. The *t*-test was used to make a comparison between the two groups. One-way variance analysis (ANOVA) was used where there are more than two groups, followed by the Tukey multiple comparisons (GraphPad Software Inc., San Diego, CA, USA). In all cases, a *P* value of less than 0.05 was considered statistically significant.

## 3. Results and Discussion

### 3.1. Optimization Studies

**Figure 1:**
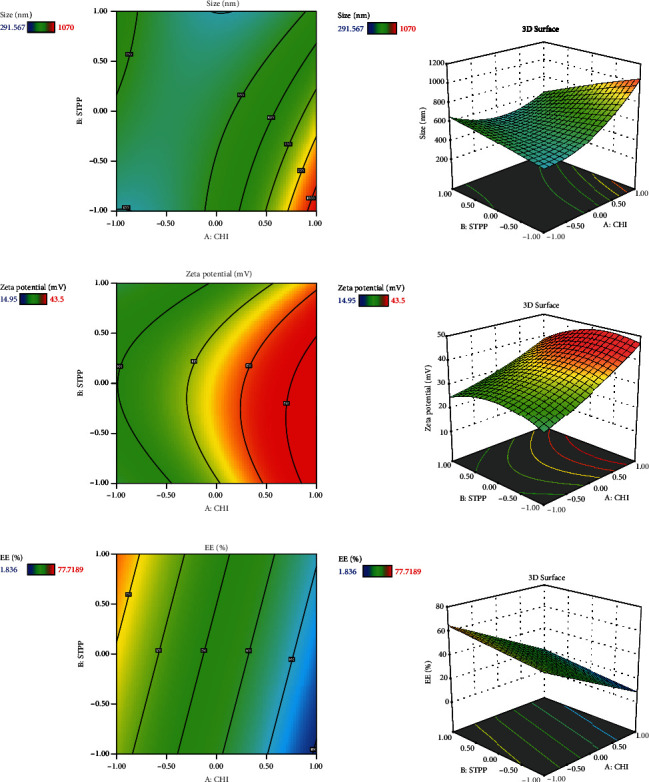
2D contour plots and 3D response surface plots presenting the effect of the independent variable on (a) size, (b) zeta potential, and (c) EE%.

Since different variables may have impacts on the responses and also in conventional methods, the influence of each variable on each response is not usually considered, and the application of RSM is necessary. This method is aimed at decreasing the total experiments required for the study while enhancing the results by involving a variety of variables and investigating their impact on the selected responses [37]. RSM has a variety of experimental designs such as a three-level factorial design, Box-Behnken design, central composite design, and Doehlert design [41]. In the current study, the Box-Behnken design was conducted to investigate the effect of CHI (*A*, mg/ml), STPP (*B*, mg/ml), and HA (*C*, mg/ml) on the size (nm), PDI (value), zeta potential (mV), and entrapment efficiency percentage (EE%) as dependent responses with a total number of 15 formulations. The analysis of variance (ANOVA), 3D plots (Figure 1), regression coefficients, and regression equation were obtained by using Design-Expert software (version 11, Stat-Ease Inc., Minneapolis, USA). The specific details of the variables from the ANOVA findings are exhibited in Tables 3–5. CHI, HA, and STPP concentrations were selected as independent variables based on the range of previous studies (Table 1) [42–44].

CHI and gelatin are natural components that were employed in the present study to prepare MET-loaded NPs. Due to their biocompatibility and biodegradability, they have gained the attention of many researchers [45, 46]. CHI has several amino groups which could be protonated in an acidic medium and form positive charges. These positive charges could have interactions with a countercharge of polyanions from gelatin [45, 46]. The ionic gelation method has been used to encapsulate drugs by forming complexes by electrostatic and hydrogen bonds from natural products. However, the method of preparation and the physical conditions have a significant effect on the properties of CHI NPs. In a study performed by Rampino et al., it was reported that the optimum pH value for the preparation of nanoparticles should be 5 [47]. Also, in a similar study conducted by Nazeri et al. [42], they showed that to prepare CHI and HA NPs by ionic gelation, the best pH to obtain the smallest particle size with a maximum zeta potential was between 5 and 5.5. They also showed that the particle size increased when steering time was decreased from 180 to 60 seconds, but in contrast, the zeta potential value increased [42].

### 3.2. The Effect of Variables on NP Size

The physicochemical characteristics of NPs strongly depend on the formulation composition.  Table 6 shows the size, PDI, zeta potential, and EE% values for each formulation. The results for observed responses were tested using ANOVA and presented in Table 3. The *F* value and *P* value are 7.14 and 0.0217, respectively, which demonstrates the significance of the quadratic model (Table 3). Therefore, this model was selected for the actual response surface. The equation below shows the relationship between variables and NP size:

(3) $$\begin{array}{c} \text{Size}=573.28+129.63A-65.39B+83.07C-145.06AB-5.17AC+57BC+143.7A^{2}-9.12B^{2}-89.71C^{2}. \end{array}$$

Based on the results revealed by ANOVA (Table 3), the particle size is dependent on the amount of CHI, STPP, and HA (*P* < 0.05); in fact, as the amounts of CHI and HA increase, the size of NPs increases accordingly. A study by Agarwal et al. showed that the optimum concentration of CHI to make polymeric NPs below 500 nm was 4 mg/ml and for STPP a concentration below 1.5 mg/ml [44]. This could be due to the formation of inter- and intramolecular crosslinks provided by the STPP (a polyanionic cross-linker), which eases the aggregation of NPs more rapidly [47]. In fact, STPP can be functionalized as a stabilizer of the prepared polymer network. It increases the compactness of the network structure. However, lowering its concentration can cause the network to lose its shape [48]. The possible interactions between positive and negative charges of NPs may cause aggregation. The aggregation can be formed as a result of interaction among oppositely charged NPs or when there is no electrostatic stability during the manufacturing of nanoparticles [49]. The influence of HA might be due to the interaction of negative charge on the surface, which can also impact the zeta potential value [50].

In a study carried out by Fan et al. on CHI NPs, the relationship between size enlargement and CHI content was noted, but the size was not directly related to the amount of STPP [51]. In another research carried out by Koukaras et al., the optimum ratio of CHI to STPP was 4 : 1 which produced the smallest particle size (340 nm), which is in agreement with the results obtained in this study [52].

### 3.3. The Effect of Variables on the PDI

PDI was not significantly associated with any of the independent variables based on the available data (*P* > 0.05). This result is in contrast with Nazeri et al.'s study that predicted the lowest values of PDI obtained when the lowest ratio of CHI and STPP was used [42]. However, according to the studies of Nazeri et al. and Kalam, the PDI depends mostly on the duration of stirring, the rate of stirring per minute, and the sonication time. Therefore, to keep this response optimal, it seems that more attention should be made to the conditions and the method of making NPs rather than paying attention to the amounts of ingredients [42, 53].

### 3.4. The Effect of Variables on Zeta Potential (mV)

The ANOVA findings of the *F* value and *P* value for the polydispersity index are 7.83 and 0.0178, respectively, which demonstrate that the best-fitted model is quadratic (Table 4). There is a significant relationship between the amount of CHI and zeta potential value (*P* ≤ 0.05) that zeta potential increases with the addition of chitosan level. The equation below implies the relationship between zeta potential and the variables:

(4) $$\begin{array}{c} \text{Zeta potential}=37.44+9.01A-2.04B+1.97C-1.94AB-1.10AC-7.53BC+1.5A^{2}-4.88B^{2}-9.68C^{2}. \end{array}$$

This may be related to the effect of CHI with its amino groups, which protonate and induce more positive charges on the surface [42]. Although the value of zeta potential was good and acceptable in all the prepared formulations, during the selection of the optimal formulation by the statistical method, the absolute value of zeta potential should be high to ensure suitable stability of the formulation. In addition, the positive zeta potential of the formulation can give rise to a strong electrostatic interaction with the negative charge of skin cells, which can increase the cellular uptake of MET [22]. HA and STPP contents are not significantly related individually, which is relatively consistent with the studies of Chiesa et al. [54]. However, the negative charge of HA could interfere with the effective electric charge on the NP's surface and impact the reduction of the zeta potential value, according to Dhayanandamoorthy et al. [50].

### 3.5. The Effect of Variables on the Entrapment Efficiency (EE%)

*F* value and *P* value for EE% are 9.79 and 0.0019, respectively, and the best-fitted model is linear according to the data reported in Table 5. The rate of drug encapsulation in NPs is significantly related to the value of CHI (*P* ≤ 0.05). The equation below shows the relation between the variables and EE%.

(5) $$\begin{array}{c} \text{EE}\%=37.14-22.20A+5.79B-8.14C. \end{array}$$

The amount of drug encapsulated in NPs depends on the CHI to drug ratios, the cross-linker (STPP) level, and the electrostatic interactions between the polymeric matrix of the NPs [42, 55]. This may be due to the presence of crosslinking agent with its capability to enhance the presence of the drug in its structure [6]. As the amount of CHI increases, there would be more interactions with the carboxylic acid groups of gelatin, which also could be a reason to improve EE% [31]. Furthermore, the presence of gelatin in the preparation of colloidal drug delivery systems may help to increase the surface charge or zeta potential and also can increase drug encapsulation performance, which leads to improved bioavailability [56–59]. In a recent study conducted by Esteban-Pérez et al., the inclusion of timolol maleate in gelatin NPs boosted bioavailability compared to its formulation in the marketed [60]. Also, in another study, researchers obtained high EE% while using HA in the preparation of tocopherol and cholecalciferol-loaded NPs [48]. In another study conducted by Sobhani et al., it was shown that when the content of ciprofloxacin in the CHI : drug ratio increased, the EE of NPs decreased [43].

### 3.6. Optimization and Validation of the Model

When optimizing a nanoformulation, the researchers usually consider the conditions to obtain small particle size, highest absolute zeta potential value, and highest EE%. Based on the intended range of response values, independent components were derived through statistical and graphical analyses. To choose an optimum formulation, the Design-Expert software point prediction was applied based on the desirability factor close to 1.

The selected formulation had 1.417 mg/ml CHI (*A*), 0.3 mg/ml STTP (*B*), and 0.2 mg/ml HA (*C*). For the above composition, the response variable predicted values were within the required range (average particle size 323.76 nm, zeta potential 22.15 mV, and percent EE 64%). The checkpoint's experimental sample was produced and characterized for the response variables using the projected independent factors. The actual outcomes (average particle size of 329 ± 6.30 nm, zeta potential of 21.94 ± 0.05 mV, and EE of 64.71 ± 6.12%) were in excellent accordance with the predicted values by the software.

### 3.7. DSC Analysis

**Figure 2:**
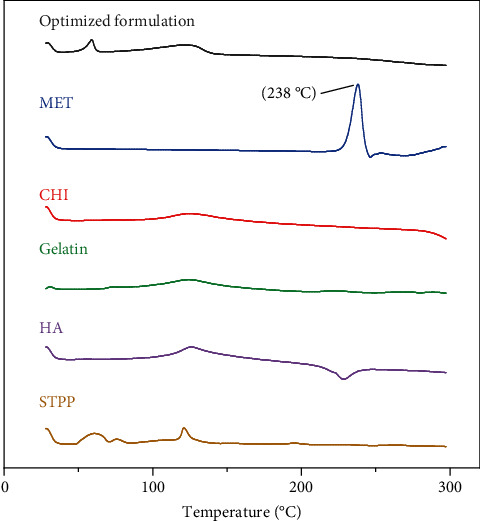
DSC thermogram of STPP, HA, gelatin, CHI, MET, and optimized formulation.

Figure 2 illustrates the thermal analysis of MET, CHI, gelatin, HA, STPP, and NPs. As it is obvious from Figure 2, the MET thermoplastic peak is at 238°C and HA has a sharp exothermic peak at 235°C, which implies that both of these substances are in their crystalline state. In the thermogram of the NPs, neither of these peaks is exhibited; this may be because these components are transformed from crystalline structure to amorphous form. MET and HA are molecularly embedded in the structure of the NPs and are no longer present in their original crystalline form [31].

### 3.8. ATR-FTIR Results

**Figure 3:**
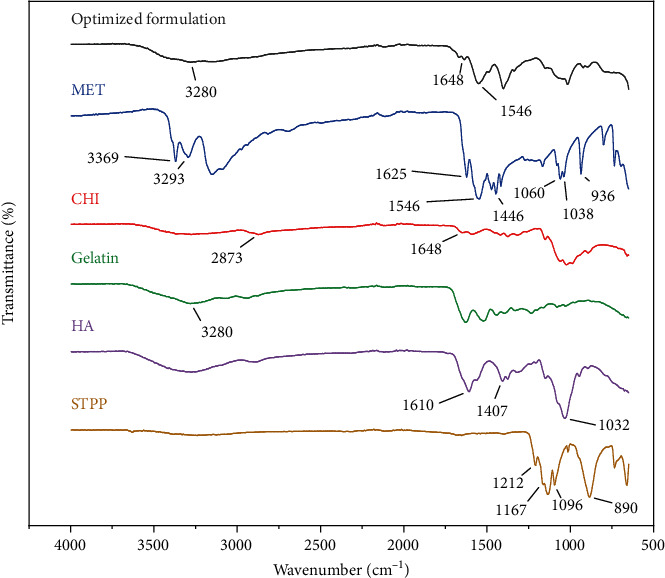
The ATR-FTIR spectra of STPP, HA, gelatin, CHI, MET, and optimized formulation.

ATR-FTIR spectra of MET, gelatin, CHI, HA, STPP, and NPs are displayed in Figure 3. According to Figure 3, MET spectrum peaks are as follows: 3369 cm^−1^ (N-H stretching), 3293 cm^−1^ (second N-H stretching), 1546 cm^−1^ (C=N stretching), and 1446 cm^−1^ (C-H bending). It is confirmed that the sharp peaks in the range of 3369 and 3293 cm^−1^ disappeared and a wide peak was observed in the 3281 cm^−1^ area, which is probably related to the overlap of the MET bonds and gelatin peaks. Also, sharp peaks are visible in 1546 cm^−1^ which may be related to the same (C=N stretching) bonds of MET. It could be concluded that the diagnostic peaks of MET crucial peaks are still recognizable in the NP spectra. CHI shows stretching, symmetric and asymmetric peaks at 2873 cm^−1^ and 3200 cm^−1^, related to the first amine group, and a peak at 1648 cm^−1^ related to the hydroxyl group, respectively. This peak is also observed in the spectra of the optimum formulation of NPs with a slight overlap. It should be noted that the optimum formulation also contains peaks of gelatin at 3280 cm^−1^. The STPP spectra revealed distinctive functional group PO_2_ and P=O stretching vibration peaks at 1167 and 1212 cm^−1^, respectively. P-O-P stretching vibration was detected at 890 cm^−1^. The HA spectra exhibited sharp peaks at 1610 and 1407 cm^−1^ related to asymmetrical C=O stretching and symmetrical C-O stretching of the -COO- group, respectively. Also, the stretching of the groups C-O-C, C-O, and C-O-H resulted in a significant peak at 1032 cm^−1^. According to the mentioned information, it could be concluded that all the materials utilized in the preparation of the NPs are correctly placed in the structure of NPS and have not been destroyed [31].

### 3.9. Morphology Images and Size Distribution of the Optimized Formulation

**Figure 4:**
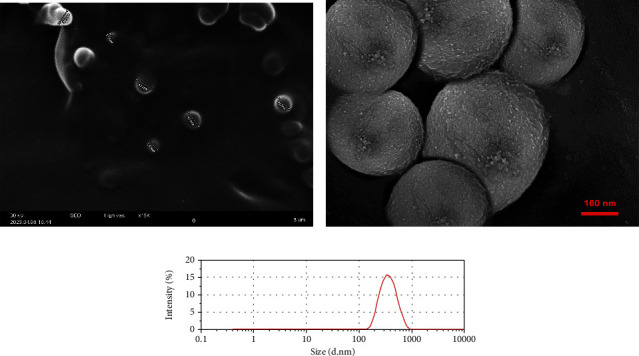
(a, b) SEM photographs of the optimized formulation. (c) Size distribution diagram of the optimized formulation.

SEM image is depicted in Figure 4. As the typical morphology of optimized formulation demonstrates, the particles were segregated and spherical and have uniform surfaces. The size distribution of the optimized formulation is also shown in Figure 4(c). The size diagram indicates the acceptable average size of NPs for skin delivery of drugs. This is correlated with the previous studies that suggest NPs between 50 and 500 nm can enhance permeation of the epidermis through the SC [61].

### 3.10. CTAB Turbidimetric Analysis

CTAB turbidimetric assay was conducted to confirm the existence of HA in NPs. The results showed that about 55% of HA existed in the optimized formulation of the NPs.

### 3.11. *In Vitro* Release Study

**Figure 5:**
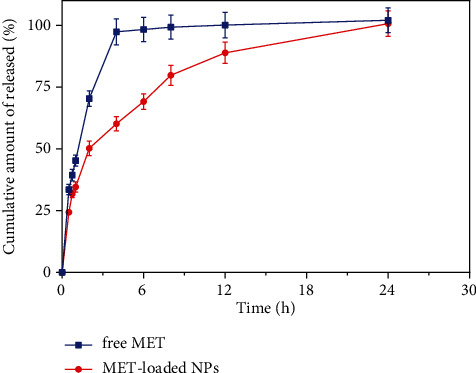
The release profile of optimized formulation and drug solution (free MET) (data are shown as mean ± SD, *n* = 3).

Figure 5 depicts the *in vitro* release pattern of the optimized formulation and free drug solution. According to Figure 5, the optimized formulation demonstrates a biphasic *in vitro* release behavior characterized by an initial burst release within the first 2 h, followed by slower and persistent release rates over 24 h than the drug solution (*P* < 0.05). This may be related to the entrapment of the drug in a network of polymers that do not allow it to be released rapidly [26]. The kinetic study showed that the Korsmeyer–Peppas model was the most compatible kinetic model that can translate MET release patterns from optimized formulation (Table 7). In this model, the *n* value (diffusional exponent in the Korsmeyer–Peppas model) was evaluated to be 0.436 for the optimized formulation, which means that the Fickian diffusion is the main mechanism of drug release. The Higuchi and Korsmeyer–Peppas models were highly consistent with the drug release profile, indicating that MET molecules were released from the dialysis membrane by the Fickian diffusion.

### 3.12. *Ex Vivo* Skin Permeation and Retention Study

**Figure 6:**
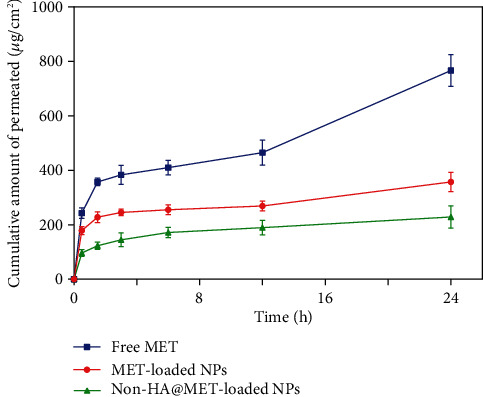
Permeation profile of MET from the investigated formulation across rat skin. Data were presented as the mean ± SD of three rats.

**Figure 7:**
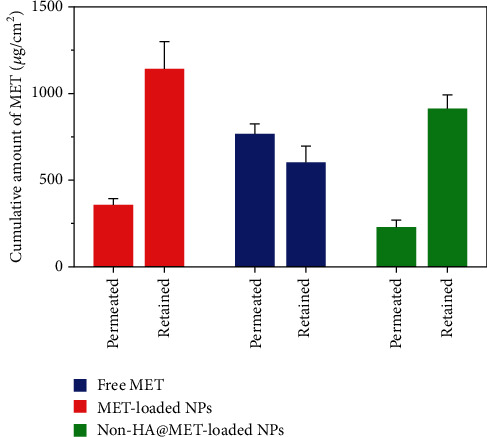
The incremental amount of MET retained (dermal delivery) and permeated through rat skin (transdermal delivery). Data were presented as the mean ± SD of three rats.

*Ex vivo* skin permeation experiments of the optimized MET-loaded NPs, non-HA@MET-loaded NPs, and free MET solution were performed at time intervals of 0.5, 1.5, 3, 6, 12, and 24 h. The results of the cumulative data after 24 h declared that the amount of drug permeated through the skin layers (transdermal delivery) was about 357.4 ± 35.7 *μ*g/cm^2^ and 228.7 ± 41.3 *μ*g/cm^2^ for MET-loaded NPs and non-HA@MET-loaded NPs, respectively. This value for the MET solution was about 766.7 ± 58.2 *μ*g/cm^2^, approximately two times higher than the MET-loaded NPs (*P* < 0.0001). The amount of drug permeated through the skin is higher in the MET-loaded NP formulation compared to the non-HA@MET-loaded NP formulation (*P* < 0.05). The amounts of MET retained in the skin layer (dermal delivery) were 1142.5 ± 156.3 *μ*g/cm^2^ and 912.3 ± 79.6 *μ*g/cm^2^ for MET-loaded NPs and non-HA@MET-loaded NPs, respectively. Despite lower skin permeation of the optimized MET-loaded NP formulation compared to the MET solution (Figure 6), a higher amount of MET was retained in the skin layer when the optimized MET-loaded NP formulation was compared to the MET solution (603.2 ± 93.1 *μ*g/cm^2^) (Figure 7), indicating the less possibility of systemic absorption and consequently less side effect of optimized formulation. Hair follicles are an important pathway for improving skin penetration for both transdermal and dermal administrations. Regardless of NP shape, the mean size of NPs is the most important indicator influencing the amount of follicular penetration [62]. Positively charged NPs can improve skin penetration by interacting with negatively charged SC [63]. To test this notion, prednicarbate-loaded nanoemulsions with both positive and negative charges were tested *in vitro* and *ex vivo* on the skin to assess the skin penetration of the obtained nanoemulsions with different charges [64]. The results indicated that while negatively charged nanoemulsions released much more drugs than positively charged nanoemulsions, positively charged nanoemulsions had significantly more skin penetration. An earlier study suggests that quaternized CHI-coated NPs with a positively charged structure might be a feasible transdermal delivery method for therapeutic ingredients [65]. The increased positive charge of this type of nanoparticle may result in stronger electrostatic attraction with the negative charge of skin bioactive molecules, as well as electrostatic deposition in layers of the skin. Walunj et al. have created and analyzed immunosuppressive drug-loaded cationic liposomes for antipsoriasis impacts. The positive charge on the surface of NPs exhibited a strong attraction for the anionic skin membrane, resulting in increased medication efficacy [66]. Similarly, Park et al. investigated the influence of zeta potential on resveratrol absorption through the skin [67]. Resveratrol penetration through the skin from chitosan-coated liposomes (zeta potential +26.5 mV) was significantly greater than that from uncoated liposomes (zeta potential -9.4 mV). As mentioned before, CHI with its positive charge could have interventions with negatively charged lipids of the SC, leading to better MET deposition. HA is a hygroscopic polymer that is capable of retaining water up to 1000 times its own weight [68]. HA may hydrate both the SC and the dermis because of its excellent water-absorption capability [69]. As a result of the significant hydration, corneocytes can swell, intercorneocyte dissociation occurs, lipid self-assembly can change structurally, and ultimately, the hydrated skin can be more permeable which is consistent with the permeation study [69]. The *α*-helix arrangement of keratin is interconverted into *β*-sheet structure once HA is connected to the SC, and the creation of *β*-sheet structures in keratin can lead to reduced barrier integrity [70]. In a study by Son et al. on transdermal delivery of lipophilic substances, drug-loaded nanohydrogel was detected in the SC, epidermis, and dermis after 24 h of administration, in contrast to the free drug without HA, which was only visible in the top layer of the epidermis [71]. In addition, the capability of HA to have an occlusive effect because of its polymeric chain involved with skin and demonstrates its hygroscopic features which could be another reason that improves MET skin deposition [22]. In a study by Asgarirad et al., they also found better histopathological results due to better accumulation of CHI NPs into the skin in comparison to the drug solution [26].

### 3.13. *In Vitro* Cytotoxicity Assay in Melanoma Cells

**Figure 8:**
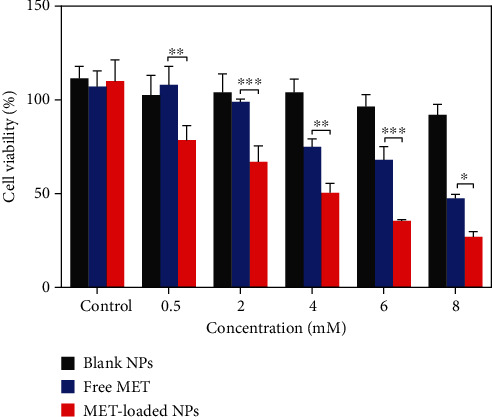
Cytotoxicity analysis of A375 melanoma cancer cells after treatment with blank NPs, free MET, and MET-loaded NPs. The results are shown in mean ± SD, *n* ≥ 3. The statistical significance is expressed as ^∗∗∗^*P* < 0.001, ^∗∗^*P* < 0.01, and ^∗^*P* < 0.05.

With the relevance of cytotoxic agents and cell death assays in cancer therapy development in mind, the cytotoxic activity of MET-loaded NPs, blank NPs, and free MET on the A375 melanoma cell line was evaluated. After 24 h, MET and its nanoformulation reduced cell viability in a concentration-dependent manner (Figure 8). This was in agreement with previously published results indicating MET dose-dependent cytotoxic effects against cancer cells [72–74]. As seen, blank NPs have no cytotoxicity effect compared to other formulations, and cell viability remained more than 90% even at the highest concentration tested. Furthermore, the total cell cytotoxic/death effects of MET-loaded NPs (3.94 ± 0.57 mM) and free MET (7.63 ± 0.26 mM) were detected (in terms of respective IC_50_ values). MET-loaded NPs had a much stronger and more significant impact on melanoma cell lines (*P* < 0.05) than free MET (Figure 8), indicating that MET has a greater cytotoxic activity when utilized as a nanoformulation. All these results suggest a better cytotoxic potential of HA-coated CHI/gelatin NPs than free MET against the A375 cell lines. A few researches have shown that HA-modified NPs attach more easily to the melanoma cell line with higher expression of CD44 receptors [75]. This behavior may also be attributed to the fact that the membrane surface of A375 cells expresses a massive amount of CD44 molecules [76, 77]; thus, the HA-modified MET-loaded NPs can bind to A375 cells more readily. It is expected that the increased cytotoxic effects of MET-loaded NPs might be because of the higher cellular uptake and sustained release of MET from internalized MET-loaded NPs that maintained a high intracellular drug concentration within the A375 cells. MET causes melanoma cell death through both AMPK-dependent and AMPK-independent mechanisms [78]. MET triggers cell cycle arrest in melanoma cells, which occurs in the induction of autophagy and, as a result, the initiation of apoptosis, which leads to melanoma cell death [79]. Furthermore, most cancerous cells possess constitutively active NF-*κ*B, which is necessary for their survival [80]. By suppressing the NF-*κ*B pathway, MET may reduce NF-*κ*B signaling pathway products, reducing cell transformation and proliferation [78, 81].

### 3.14. Annexin V/PI Staining

**Figure 9:**
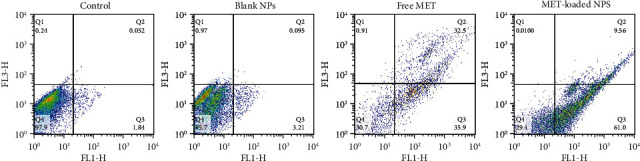
Apoptosis induction in A375 cells was treated with IC_50_ concentrations of MET-loaded NPs and free MET for 48 h and analyzed using flow cytometry.

Using Annexin V-FITC/PI staining, the percentage of apoptotic and dead A375 cells was assessed in order to determine the reason for the proliferation inhibitory mechanism resulting from MET-loaded NPs and free MET. The findings presented in Figure 9 indicate that incubating A375 cells with MET promotes phosphatidylserine translocation from the interior membrane of the cells to the outside membrane surface, which is identifiable by Annexin V and is potentially attributable to processes associated with apoptosis [82]. The graph is divided into four sections (Q), with Q1 representing necrotic cells, Q2 and Q3 representing late and early apoptotic cells, respectively, and Q4 indicating viable cells. The signs of apoptosis were detected in cells treated with MET-loaded NPs and free MET; however, the proportion of early and late apoptotic cells differed with the formulation. The fraction of cells in the Q3 section, which correspond to early apoptotic cells, rose to 61.0% and 35.9%, respectively, after exposing melanoma cells to MET-loaded NPs and free MET. The proportion of cells in the Q2 section of late apoptosis increased to 9.56% and 32.5% after treating the melanoma cells with MET-loaded NPs and free MET, respectively. There is growing research suggesting that MET may directly reduce the proliferation of cells in a variety of tumors, including the ovarian, colon, and breast [83–85]. Faramarzi et al. also showed that MET-loaded PLGA-PEG NP delivery caused cellular death in SKOV3 ovarian carcinoma cells. They reported that free MET and MET-loaded PLGA-PEG NPs raised the proportion of cellular death by 23.67% and 45.35%, respectively, in comparison to the control [86]. The observations confirm the theory that cell proliferation and apoptosis are linked to increased endocytosis and a different release profile in response to MET formulation.

## 4. Conclusion

In the present research, NPs were successfully prepared by an ionic gelation method and were optimized by RSM. This research indicated that NPs of MET and natural polymers could be suitable carriers for skin delivery of the drug. The involvement of CHI and HA could increase the delivery of the drug to the skin. In order to have a better comprehension of the effect of variables on the responses, RSM in terms of BBD (Box-Behnken design) was conducted. In conclusion, NPs prepared with natural polymers with their unique characteristics may have a superior influence on enhancing percutaneous absorption than simple drug solutions which present the potential of MET NPs for further *in vivo* investigations to treat skin diseases such as melanoma.

## Figures and Tables

**(a):** 

Independent variable	Symbol	Coded levels		
		-1	0	+1
CHI (mg/ml)	A	1	2	3
STPP (mg/ml)	B	0.1	0.3	0.5
HA (mg/ml)	C	0.2	0.6	1

**(b):** 

Dependent variables (responses)	Units	Goal
*Y*_1_: mean vesicle size	nm	Minimize
*Y*_2_: zeta potential	mV	Maximize
*Y*_3_: EE%	Percentage	Maximize

**Table 2:** Composition of independent variables of the Box-Behnken design.

Formulations^∗^	CHI (mg/ml)	STPP (mg/ml)	HA (mg/ml)
F1	3	0.3	0.2
F2	2	0.5	0.2
F3	1	0.3	0.2
F4	2	0.3	0.6
F5	2	0.3	0.6
F6	3	0.3	1
F7	1	0.1	0.6
F8	2	0.1	1
F9	3	0.1	0.6
F10	3	0.5	0.6
F11	2	0.5	1
F12	1	0.5	0.6
F13	1	0.3	1
F14	2	0.3	0.6
F15	2	0.1	0.2

^∗^The amount of MET (10 mg) and gelatin (5 mg) was constant in all formulations.

**Table 3:** Particle size statistical analysis result.

Source	Sum of squares	df	Mean square	*F* value	*P* value	
Model	435700.00	9	48409.92	7.14	0.0217	Significant
*A*-CHI	134400.00	1	134400.00	19.83	0.0067	
*B*-STPP	34204.20	1	34204.20	5.05	0.0746	
*C*-HA	55200.57	1	55200.57	8.14	0.0357	
*AB*	84167.68	1	84167.68	12.42	0.0169	
*AC*	106.78	1	106.78	0.0158	0.9050	
*BC*	12996.00	1	12996.00	1.92	0.2248	
*A* ^2^	76244.12	1	76244.12	11.25	0.0202	
*B* ^2^	306.94	1	306.94	0.0453	0.8399	
*C* ^2^	29714.71	1	29714.71	4.38	0.0905	
Residual	33896.26	5	6779.25			
Lack of fit	28490.97	3	9496.99	3.51	0.2294	Not significant
Pure error	5405.29	2	2702.65			
Cor total	469600.00	14				

**Table 4:** Statistical analysis result of zeta potential.

Source	Sum of squares	df	Mean square	*F* value	*P* value	
Model	1394.34	9	154.93	7.83	0.0178	Significant
*A*-CHI	650.10	1	650.10	32.85	0.0023	
*B*-STPP	33.35	1	33.35	1.69	0.2509	
*C*-HA	31.01	1	31.01	1.57	0.2660	
*AB*	15.02	1	15.02	0.7588	0.4235	
*AC*	4.84	1	4.84	0.2446	0.6419	
*BC*	226.75	1	226.75	11.46	0.0196	
*A* ^2^	8.30	1	8.30	0.4194	0.5458	
*B* ^2^	87.93	1	87.93	4.44	0.0889	
*C* ^2^	345.68	1	345.68	17.47	0.0087	
Residual	98.94	5	19.79			
Lack of fit	64.75	3	21.58	1.26	0.4706	Not significant
Pure error	34.20	2	17.10			
Cor total	1493.28	14				

**Table 5:** Statistical analysis result of EE%.

Source	Sum of squares	df	Mean square	*F* value	*P* value	
Model	4741.49	3	1580.50	9.79	0.0019	Significant
*A*-CHI	3943.96	1	3943.96	24.42	0.0004	
*B*-STPP	267.76	1	267.76	1.66	0.2243	
*C*-HA	529.78	1	529.78	3.28	0.0975	
Residual	1776.65	11	161.51			
Lack of fit	1732.55	9	192.51	8.73	0.1069	Not significant
Pure error	44.09	2	22.05			
Cor total	6518.14	14				

**Table 6:** Physicochemical characteristics of HA-coated CHI/gelatin NPs containing MET.

Formulations	Size (nm)	PDI (value)	Zeta potential (mv)	EE (%)
F1	598.50	0.507	40.63	25.28
F2	291.57	0.272	23.43	56.78
F3	408.90	0.402	16.60	77.72
F4	521.03	0.305	36.40	36.00
F5	573.80	0.404	33.93	29.18
F6	835.30	0.434	39.73	28.13
F7	440.63	0.242	25.40	42.88
F8	543.33	0.386	37.40	29.37
F9	1070.00	0.350	43.50	1.84
F10	684.97	0.480	38.85	21.56
F11	490.70	0.308	14.95	10.14
F12	635.83	0.537	28.50	69.34
F13	666.37	0.409	20.10	64.49
F14	625.00	0.503	42.00	27.00
F15	572.20	0.555	15.77	37.46

**Table 7:** Drug release kinetic data of MET solution and MET colloidal dispersion.

	Zero order		First order		Korsmeyer–Peppas			Higuchi	
	*R* ^2^	RMSE	*R* ^2^	RMSE	*R* ^2^	*n*	RMSE	*R* ^2^	RMSE
Optimized formulation	0.778	11.585	0.639	17225	0.976	0.436	5.067	0.513	17.138
MET solution	0.981	3.227	0.934	6.421	0.994	0.415	1.388	0.992	2.121

*R* ^2^: linear regression coefficient.

## Data Availability

Datasets used and/or analyzed during the current study are available from the corresponding authors on reasonable request.
